# Periostin—An inducer of pro-fibrotic phenotype in monocytes and monocyte-derived macrophages in systemic sclerosis

**DOI:** 10.1371/journal.pone.0281881

**Published:** 2023-08-02

**Authors:** Mao Suzuki, Yasushi Ototake, Asami Akita, Miho Asami, Noriko Ikeda, Tomoya Watanabe, Miwa Kanaoka, Yukie Yamaguchi

**Affiliations:** Department of Environmental Immuno-Dermatology, Yokohama City University Graduate School of Medicine, Yokohama, Japan; Helmholtz-Zentrum Munich, GERMANY

## Abstract

Enhanced circulating blood periostin levels positively correlate with disease severity in　patients with systemic sclerosis (SSc). Monocytes/macrophages are predominantly associated with the pathogenesis of SSc, but the effect of periostin on immune cells, particularly monocytes and macrophages, still remains to be elucidated. We examined the effect of periostin on monocytes and monocyte-derived macrophages (MDM) in the pathogenesis of SSc. The modified Rodnan total skin thickness score in patients with dcSSc was positively correlated with the proportion of CD80^-^CD206^+^ M2 cells. The proportion of M2 macrophages was significantly reduced in rPn-stimulated MDMs of HCs compared to that of SSc patients. The mRNA expression of pro-fibrotic cytokines, chemokines, and ECM proteins was significantly upregulated in rPn-stimulated monocytes and MDMs as compared to that of control monocytes and MDMs. A similar trend was observed for protein expression in the respective MDMs. In addition, the ratio of migrated cells was significantly higher in rPn-stimulated as compared to control monocytes. These results suggest that periostin promotes inflammation and fibrosis in the pathogenesis of SSc by possible modulation of monocytes/macrophages.

## Introduction

Systemic scleroderma (SSc) is an intractable collagen disease characterized by three main pathologies: immune abnormalities, vascular involvement, and fibrosis of organs, including the skin and lungs [1]. It is a highly heterogeneous disease with varying severity and organ involvement across patients. Although the etiology remains unclear, monocytes/macrophages are considered to play an important role in the pathogenesis of SSc. The proportion of CD14^+^ cells is significantly higher among peripheral blood mononuclear cells (PBMCs) from SSc patients compared to that of healthy controls (HCs). The number of CD163^+^ cells between collagen fibers is significantly elevated in the skin of SSc patients compared to that of HCs [2]. Furthermore, M2 macrophages are considered to be an important therapeutic target for tocilizumab, nintedanib, PDE4 inhibitors, and anti-CD20 antibody in SSc [3–6].

Periostin is an extracellular matrix (ECM) protein that is widely expressed in various organs. In the skin, an environment rich in fibroblasts, which is a major source of periostin, it is typically expressed at the epidermal-dermal junction, papillae, and around hair follicles [7]. As a matricellular protein, periostin binds to several types of integrins, which act as its receptors [8]. It is involved in allergic diseases [9], tumors [10], myocardial infarction [11], wound healing [12], and fibrosis [13, 14]. We previously showed that circulating periostin levels were elevated in SSc patients, which correlated positively with the modified Rodnan total skin thickness score (mRSS) [14]. In addition, periostin-knockout in mice inhibits bleomycin-induced fibrosis in the skin and lungs [13, 15]. Thus, periostin may be a critical regulator of fibrosis and tissue remodeling. There are eight isoforms of periostin. In particular, variant 2/4 is considered a specific biomarker for SSc because the ratio of variant 2/4 to total periostin in SSc skin fibroblasts is higher than in control fibroblasts in the presence of TGF-β. Moreover, the serum levels of variant 2/4 is positively correlated with mRSS [16]. However, the effect of periostin on immune cells, monocytes, and macrophages in particular, has not been investigated.

The present study aims to elucidate the role of periostin in the differentiation of monocyte-derived macrophages (MDMs) and their phenotype in SSc.

## Materials and methods

### Patient samples

Whole blood samples were obtained from nine Japanese patients with diffuse cutaneous (dc)SSc (men, n = 1; women, n = 8; mean age, 56.6 ± 14.0 years), who fulfilled the American College of Rheumatology preliminary classification criteria for SSc [17], and from healthy volunteers (n = 6). Patient characteristics are summarized in Table 1. Disease duration was defined as the period from disease onset to the time of blood sampling. Disease onset was defined as the date of the first non-Raynaud’s symptom attributable to SSc. Skin fibrosis was evaluated using the mRSS [18]. Interstitial lung disease (ILD) was defined as bibasilar interstitial fibrosis by chest high-resolution computed tomography. Pulmonary arterial hypertension was defined as a mean pulmonary arterial pressure of > 25 mmHg. A series of autoantibodies were identified by an enzyme-linked immunosorbent assay (ELISA; MBL, Aichi, Japan). At the time of blood sampling, two patients were receiving low-dose corticosteroids (< 10 mg/day), and one was on immunosuppressants (mycophenolate mofetil).

**Table 1:** Clinical characteristics of the nine systemic sclerosis patients.

Women, n (%)	8 (89)
Age (years, mean ± SD)	56.6 ± 14.0
Disease duration (years, mean ± SD)	6 ± 5.7
Raynaud phenomenon, n (%)	6 (67)
Modified Rodnan total skin thickness score (mean ± SD)	24.8 ± 7.9
Organ involvement, n (%)	
Digital ulcers	3 (33)
Interstitial lung disease	8 (89)
Pulmonary arterial hypertension	1 (11)
Gastrointestinal involvement	6 (67)
Autoantibodies, n (%)	
Anti-topoisomerase I antibody	2 (22)
Anti-RNA polymerase III antibody	2 (22)
Anti-nuclear antibody	2 (22)
Anti-SSA antibody	2 (22)
Anticentromere antibody	1 (11)
Treatment, n (%)	
Corticosteroids (PSL < 10 mg/day)	2 (22)
Mycophenolate mofetil	1 (11)

Written informed consent was obtained from all subjects in accordance with the Declaration of Helsinki. The study was approved by the Institutional Review Board of Yokohama City University, Yokohama, Japan (Approval No: B200800007). Medical records and samples were collected from September 10, 2020 to September 8, 2022. We had access to information that could identify individual participants during the study period.

### Cell isolation

Whole blood samples were obtained from SSc patients and HCs. PBMCs were separated by density centrifugation at 400 × *g* for 30 min using Histopaque® 1077 (Millipore Sigma, St Louis, MO, USA). CD14^+^ monocytes were further purified from PBMCs using a MACS® Cell Separation system and MACS beads (CD14 microbeads; Miltenyi Biotec, Bergisch Gladbach, Germany) according to the manufacturer’s protocol. The purity of isolated monocytes exceeded 95%, as validated by assessing the expression of CD14 using flow cytometry (S1 Fig).

### Culture and stimulation of MDMs

Monocytes (2 × 10^5^ cells/mL for quantitative real-time polymerase chain reaction (qPCR) or 5 × 10^5^ cells/mL for flow cytometry and bead-based immunosorbent assays) were seeded in 6-well multi-dishes in RPMI1640 medium (FUJIFILM Wako Pure Chemical Industries, Osaka, Japan) supplemented with 1% penicillin–streptomycin (Thermo Fisher Scientific, Waltham, MA, USA) and 10% fetal bovine serum (FBS; Biowest, Nuaillé, France), with 100 ng/mL recombinant macrophage colony-stimulating factor (M-CSF; Peprotech, Rocky Hill, NJ, USA) for 6 days. On Day 1, monocytes were treated with 10 μL/mL of phosphate-buffered saline (PBS; FUJIFILM Wako) or 1000 ng/mL human recombinant periostin (rPn; R&D Systems, Minneapolis, MN, USA). On Day 7, the macrophages were stimulated with 100 ng/mL of lipopolysaccharide (LPS; Millipore Sigma) and 20 ng/mL of interferon gamma (IFN-γ; Peprotech) to induce M1 polarization (MDM1), or 20 ng/mL of interleukin-4 (IL-4; Peprotech) and 10 ng/mL of M-CSF to induce M2 polarization (MDM2). Cells were harvested 24 h post-stimulation.

### Flow cytometry analysis

MDMs were pretreated with FITC-conjugated anti-CD68, BV421-conjugated anti-CD80 (Biolegend, San Diego, CA, USA), APC-conjugated anti-CD163 (BD Biosciences, San Jose, CA, USA), and APC-conjugated anti-CD206 (BD Biosciences, San Jose, CA, USA). Single-cell suspensions were subjected to flow cytometry analysis (FACS Canto^TM^ II; BD Biosciences). Data were analyzed using the FlowJo software version 10 (FlowJo, Ashland, OR, USA). Events were gated on live and single-cell populations. CD68^+^-cells were gated as macrophages. The percentage of CD80^+^CD163^—^, CD80^+^CD206^—^, CD80^-^CD163^+^-, and CD80^-^CD206^+^-cells among these cells was further analyzed. Appropriate fluorescently labeled isotype-matched antibodies (Abs) to irrelevant antigens were used as controls for all analyses.

### Quantitative real-time polymerase chain reaction (qPCR)

Total RNA was extracted using TRIzol® (Thermo Fisher Scientific, Rochester, NY, USA) and the Illustra^TM^ RNAspin Mini RNA Isolation Kit (GE Healthcare Life Sciences, Buckinghamshire, England). First-strand cDNA was synthesized using the High-Capacity RNA-to-cDNA Kit (Applied Biosystems, Foster City, CA, USA) in accordance with the manufacturer’s instructions. qPCR was performed in duplicate using TaqMan^®^ gene expression assays (Thermo Fisher Scientific)　or Thunderbird^®^ SYBR^®^ qPCR Mix (Toyobo, Osaka, Japan).　Gene expression levels were normalized to β-actin and compared using the 2^−ΔΔCt^ method. TaqMan^®^ probes for human *TGF-β*, *TNF-α*, *MCP-1 (CCL2)*, *ACTA2*, *EGR1*, *FN1*, *IL-6*, *PDGFRB*, and *ACTB*, were obtained from Thermo Fisher Scientific. The assay identification numbers are shown in S1 Table. The primer sets for human *AGAP2-AS1* and *ACTB* used for SYBR^®^ qPCR are summarized in S2 Table.

### Bead-based immunosorbent assay

Supernatant from samples obtained from MDM cultures were used to measure the levels of TNF-α, MCP-1, and IL-6 proteins, using the LEGENDplex™ Human Inflammation Panel 1 (Biolegend), according to the manufacturer’s instructions. Measurements are representative of the mean of three independent experiments performed in duplicate.

### Total RNA isolation, RNA sequencing (RNA-seq), and data analysis

Monocytes (5 × 10^5^ cells/mL) isolated from blood samples obtained from three HCs were seeded in 12-well multi-dishes in RPMI1640 supplemented with 1% penicillin–streptomycin and 10% FBS with 10 μL/mL PBS or 1000 ng/mL human rPn. Cells were harvested 16 h post-stimulation. Approximately 1–2 μg of total RNA was extracted from six samples (PBS control, n = 3; rPn-stimulated, n = 3) to prepare sequencing libraries with the TruSeq stranded mRNA kit (Illumina, San Diego, CA, USA). These were subsequently subjected to 100-bp paired-end RNA-seq on a NovaSeq6000 platform (Illumina, San Diego, CA, USA). RNA integrity was further evaluated using the RNA Screen Tape of TapeStation (Agilent Technologies, Santa Clara, CA, USA). All samples demonstrated an RNA integrity number > 8.0. RNA-Seq Data Analysis quality control of raw RNA-seq data was evaluated using FASTQC. Reads were aligned to the genome (NCBI reference sequence; GRCh38) using the alignment program HISAT2 [19]. Subsequently, the number of reads that mapped to each gene was counted using StringTie. Differential expression analysis between the control and stimulated samples was conducted using the DESeq2 package [20]. P < 0.05 and fold-change > 2 were considered as significant differences and were set as the threshold for differentially expressed genes (DEGs). The R package ggplot2 (version 3.3.3) was used to draw the volcano plot. Public RNA-seq data from three HCs (six samples) are accessible at GEO (GSE213614, http://www.ncbi.nlm.nih.gov/geo/).

### Monocyte migration assay

Monocyte chemoattractant activity was assessed using 24-well Transwell® plates with 5-μm-pore filters (Corning Inc., Corning, NY, USA). Monocytes (5 × 10^5^ cells/mL) isolated from the blood of four HCs were placed in the upper compartment, and 500 or 1000 ng/mL of rPn was added to the lower compartment. Cells were allowed to migrate through the filter for 2 h, harvested from both compartments, and manually counted. The percentage of migrated cells was calculated as the ratio of the cell count of the lower compartment to the total cell count of the upper and lower compartments.

### Statistical analysis

The Wilcoxon rank-sum test was used for univariable comparison of two groups. Correlation between two groups was evaluated using the Spearman’s rank correlation coefficient. One-way analysis of variance followed by Dunn’s test and two-way analysis of variance followed by Sidak’s test were used for multiple comparisons. All tests were carried out using GraphPad Prism (GraphPad Software, version 7.0; San Diego, CA, USA). P < 0.05 was considered significant.

## Results

### The proportion of M2 macrophages correlates with skin sclerosis in SSc patients

**Fig 1:**
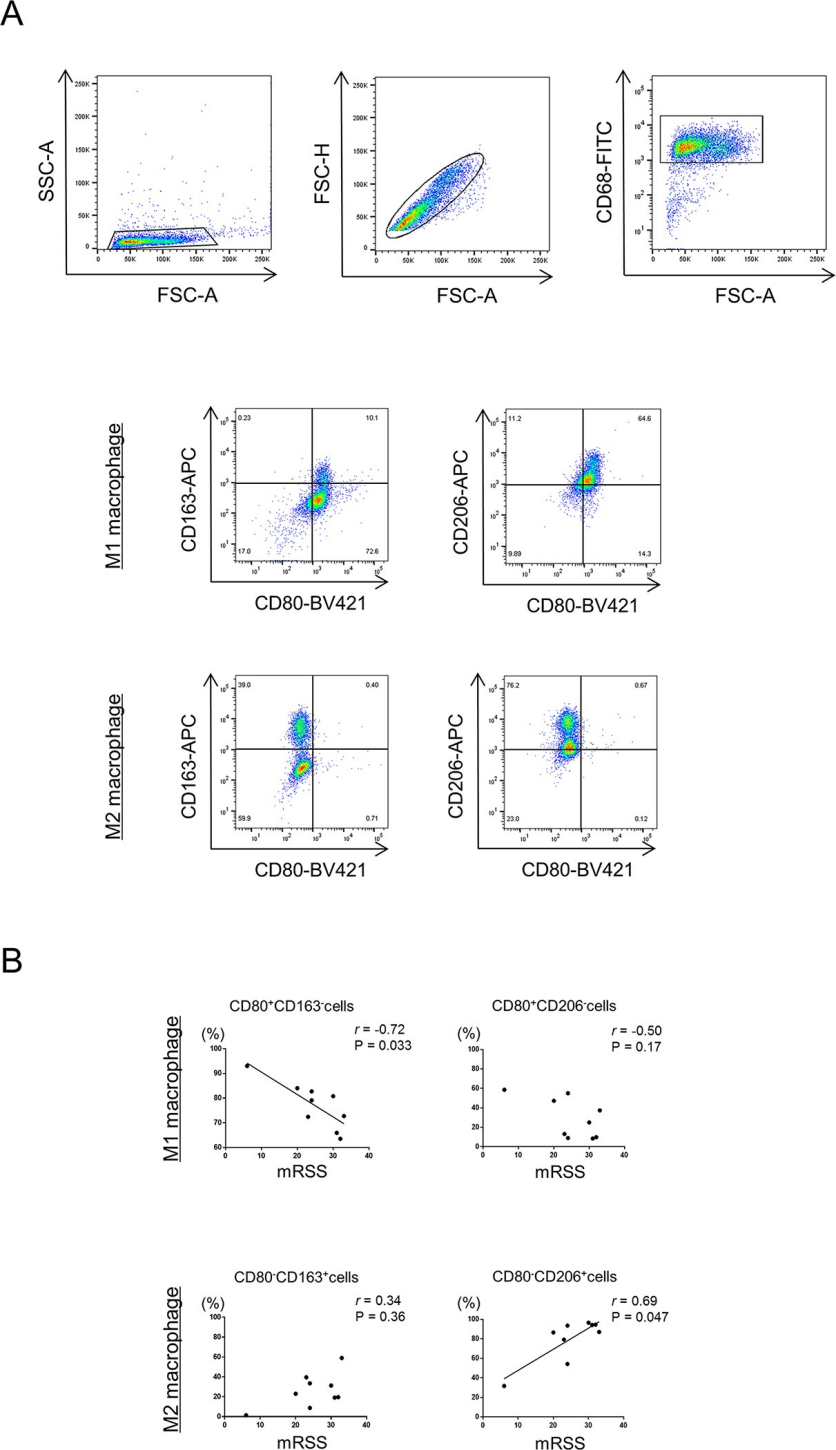
M1/ M2 polarization and modified Rodnan total skin thickness score in dcSSc patients. (A) Representative dot plots show the gating strategy for identification of CD80^+^CD163^-^, CD80^+^CD206^-^, CD80^-^CD163^+,^ and CD80^-^CD206^+^ macrophage subsets. (B) Correlations between the proportions of CD80^+^CD163^-^ or CD80^+^CD206^-^ cells in monocyte-derived macrophages (MDM) 1 and CD80^-^CD163^+^ or CD80^-^CD206^+^ cells in MDM2 to CD68^+^ cells and mRSS in patients with diffuse cutaneous systemic sclerosis (dcSSc). Correlations were determined using Spearman’s rank correlation test. Solid lines represent regression lines.

The association between polarization of MDMs and clinical features of SSc was analyzed. We first sought to analyze the proportion of cell surface markers (CD80 for M1; CD163 and CD206 for M2) of CD68^+^ macrophages by flow cytometry in SSc patients (Fig 1A). A significant negative correlation was detected between the proportion of CD80^+^CD163^-^ cells in MDM1 and mRSS, and a positive correlation between the proportions of CD80^-^CD206^+^ cells in MDM2 and mRSS (Fig 1B). No other clinical feature (disease duration and organ involvements) correlated with the proportion of MDM1/2. Thus, the more severe the skin sclerosis, the higher the proportion of M2 macrophages.

### Periostin reduces M2 macrophages in HCs but not in SSc patients

**Fig 2:**
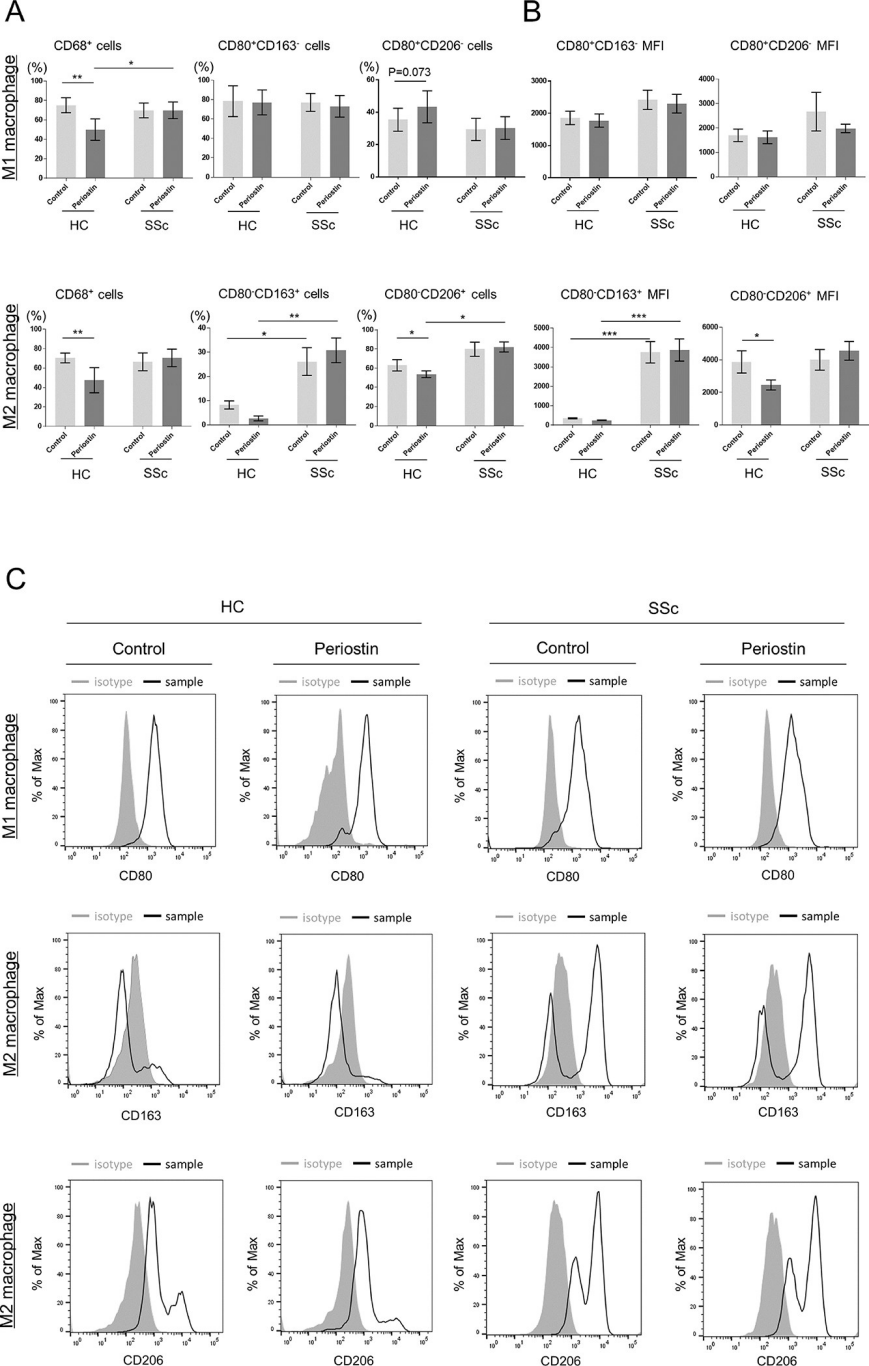
M1/M2 polarization in recombinant periostin (rPn)-stimulated MDMs. (A) Proportion of CD68^+^ cells to live and single cells in MDM1 and MDM2, CD80^+^CD163^-^ or CD80^+^CD206^-^ cells in MDM1, and CD80^-^CD163^+^ or CD80^-^CD206^+^ cells in MDM2 to CD68^+^ cells. (B) Mean fluorescence intensities (MFIs) of CD80^+^CD163^-^ or CD80^+^CD206^-^ cells in MDM1 and CD80^-^CD163^+^ or CD80^-^CD206^+^ cells in MDM2. Data are shown as the mean ± standard error of the mean (SEM). Statistical analysis was performed using the Wilcoxon rank-sum test *P < 0.05, HCs: n = 5, SSc patients: n = 9. (C) Representative histograms of the proportion of CD80 for MDM1 and CD163 and CD206 for MDM2 in HCs and SSc patients.

To investigate the effect of periostin on MDMs, monocytes were differentiated into macrophages by stimulation with rPn and further polarized into M1 or M2 macrophages *in vitro* (rPn-stimulated MDM1 or MDM2). Cell proportion and mean fluorescence intensity (MFI) are shown in Fig 2A and 2B. The population of CD68^+^ cells was significantly downregulated by rPn stimulation in HCs, but no detectable change was observed in SSc patients. The percentage of CD68^+^ cells in the rPn-stimulated MDMs from SSc patients was significantly higher than that of HCs. In addition, the proportions of CD80^-^CD163^+^ cells in both the control and rPn-stimulated MDM2 cells and the MFI were significantly higher in SSc patients than in HCs. These results revealed that there were differences in reactivity to periostin between HCs and SSc patients (MDM1: interaction effect F = 11.25, P < 0.01; MDM2: interaction effect F = 9.775, P < 0.01). In addition, rPn stimulation decreased the proportion of CD80^-^CD163^+^ cells in HCs but increased it in SSc patients (interaction effect F = 5.67, P < 0.05). rPn stimulation significantly decreased the proportion and MFI of CD80^-^CD206^+^ cells in HCs, but not in SSc patients (proportion: interaction effect F = 6.76, P < 0.05, MFI: interaction effect F = 10.24, P < 0.01). Thus, the responsiveness to periostin may differ between HCs and SSc patients. Representative histograms are depicted in Fig 2C to show whether specific cell surface markers were expressed in MDM1 and MDM2.

### rPn stimulation in MDM1/2 enhances the level of pro-fibrotic factors

**Fig 3:**
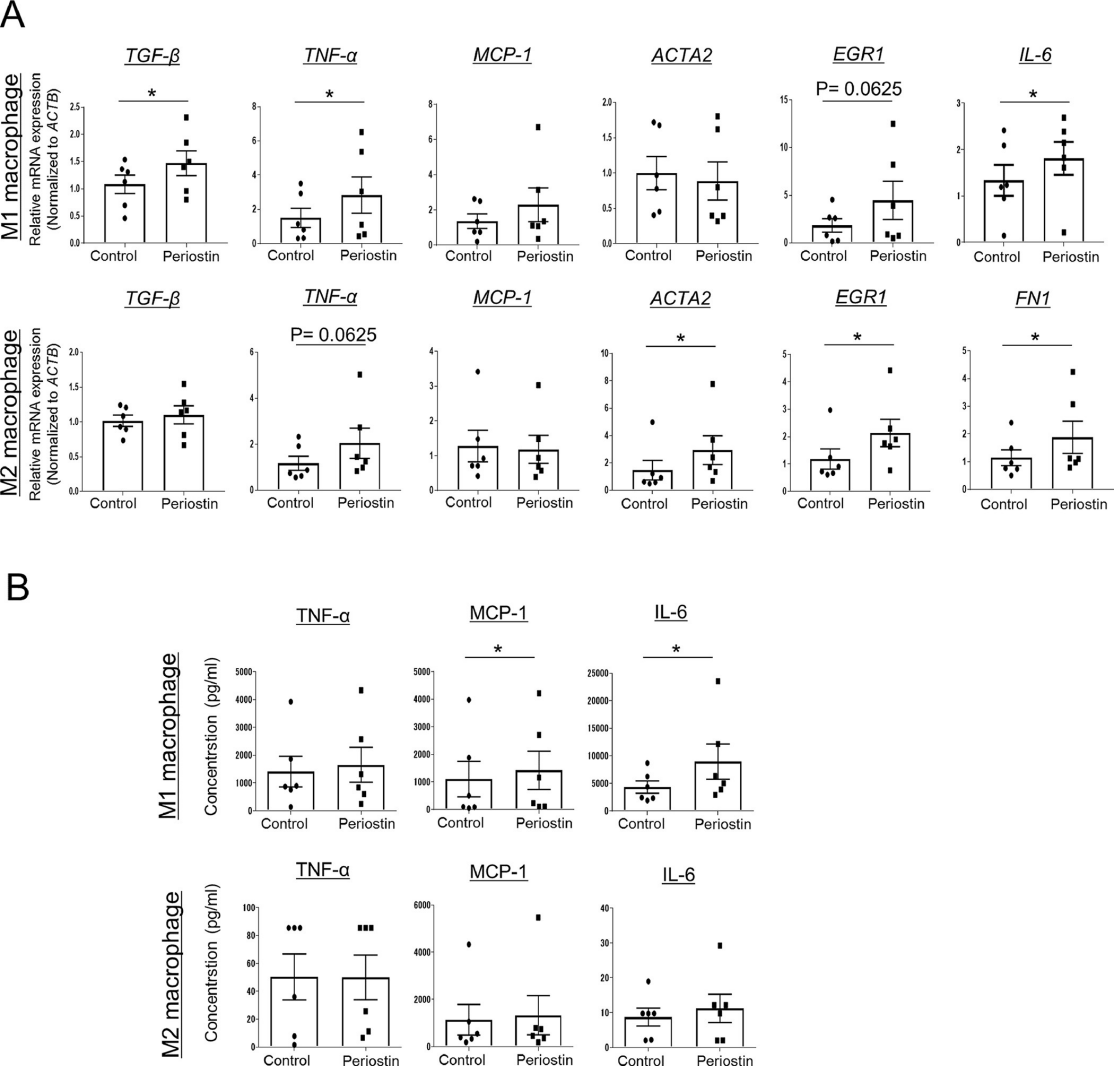
Profibrotic factors in rPn-stimulated MDMs. Function of periostin-stimulated monocyte-derived macrophages (MDMs) differentiated after isolation from six healthy controls. (A) The relative gene expression levels of *TNF-α*, *TGF-β*, *MCP-1*, *ACTA2*, *EGR1*, and *IL-6* in MDM1 and *TGF-β*, *TNF-α*, *MCP-1*, *ACTA2*, *EGR1*, and *FN1* in MDM2 were determined by quantitative real-time polymerase chain reaction. (B) Protein levels of TNF-α, MCP-1, and IL-6 in MDM1 and MDM2 measured by a bead-based immunoassay. Data are shown as the mean ± standard error of the mean (SEM). Statistical analysis was performed using the Wilcoxon rank-sum test *P < 0.05, n = 6.

The role of M1/M2 macrophages in SSc remains controversial [21, 22] because of the local micro-environment-derived polarization of macrophages. Since the function of M1/M2 macrophages is not solely assessed by cell surface markers, the gene expression profile of SSc-related cytokines, chemokines, ECM proteins, and transcription factors was evaluated using qPCR in MDM1/2 cells from six HCs. The expression of *TNF-α*, *IL-6*, and *TGF-β* was significantly higher in the rPn-stimulated MDM1 than in control MDM1. The levels of *ACTA2*, *FN1*, and *EGR1* were significantly upregulated in the rPn-stimulated MDM2 (Fig 3A). Bead-based immunoassays revealed significantly upregulated MCP-1 and IL-6 protein levels in rPn-stimulated MDM1 as compared to those in control MDM1 (Fig 3B). A similar trend was observed in rPn-stimulated MDM2, but the difference was not statistically significant. Thus, rPn-stimulated enhanced production of pro-fibrotic and pro-inflammatory factors in macrophages (M1 or M2 macrophages) may contribute to the pathogenesis of fibrosis in SSc.

### Upregulation of fibrotic and SSc-related factors in rPn-stimulated monocytes

**Fig 4:**
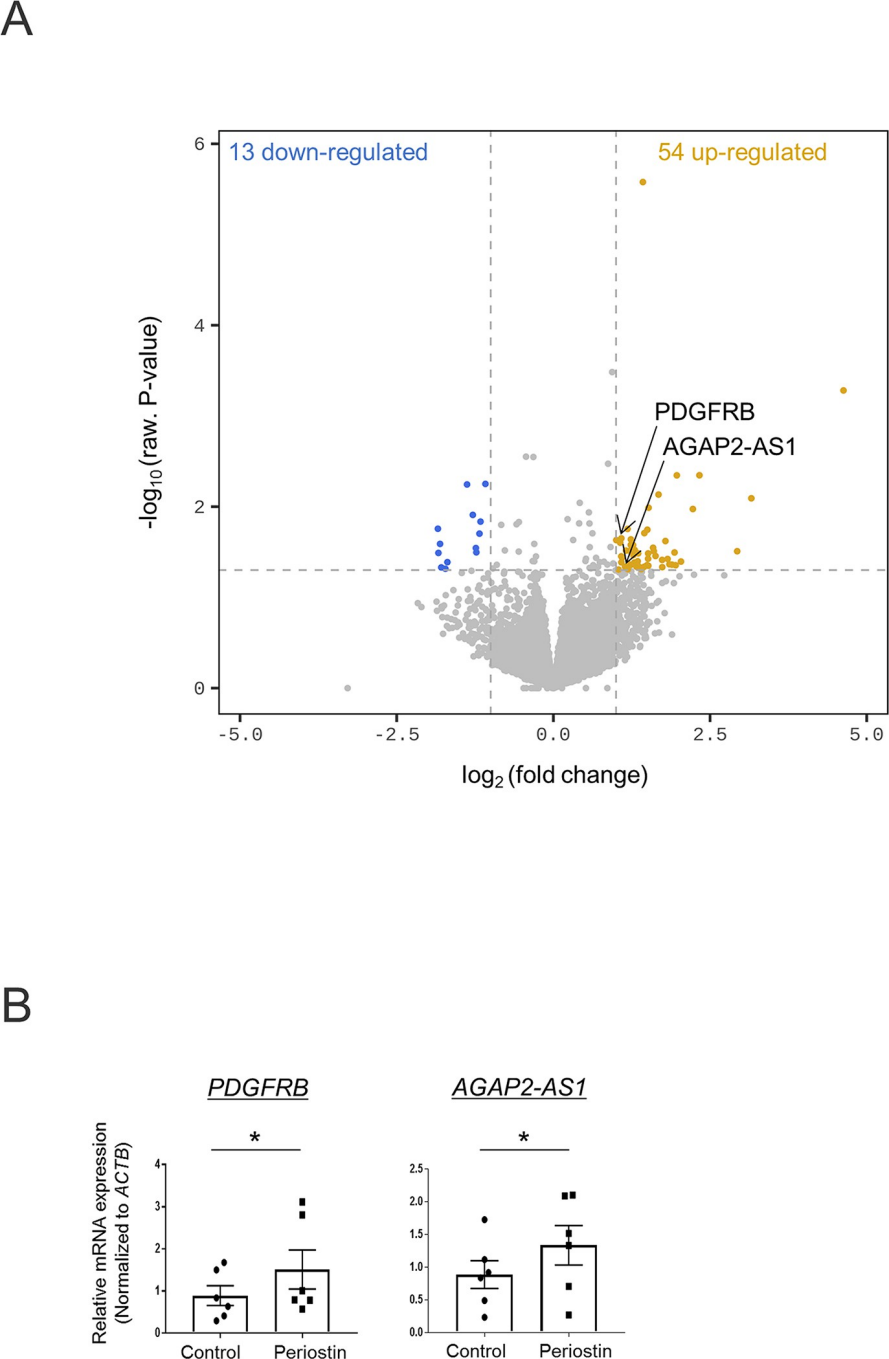
Transcriptional response in periostin-stimulated monocytes. (A) Volcano plot indicating differentially expressed genes between periostin-stimulated and control monocytes with a threshold of P < 0.05 and fold-change > 2.0. (yellow = increased, blue = decreased). (B) The relative gene expression levels of *PDGFRB* and *AGAP2-AS1 in monocytes* were determined by quantitative real-time polymerase chain reaction. Data are shown as the mean ± standard error of the mean (SEM). Statistical analysis was performed using the Wilcoxon rank-sum test *P < 0.05, n = 3.

The effect of periostin on monocytes prior to their differentiation into macrophages was evaluated using RNA-seq. Comparative analysis identified 67 DEGs between the control and rPn-stimulated monocytes (S3 Table). Based on previous studies, *PDGFRB* and *AGAP2-AS1*, reported to be involved in the pathogenesis of SSc, were chosen and subjected to qPCR analysis. Significantly enhanced expression of *PDGFRB* and *AGAP2-AS1* was seen in rPn-stimulated as compared to control monocytes (Fig 4).

### Periostin promotes monocyte migration

**Fig 5:**
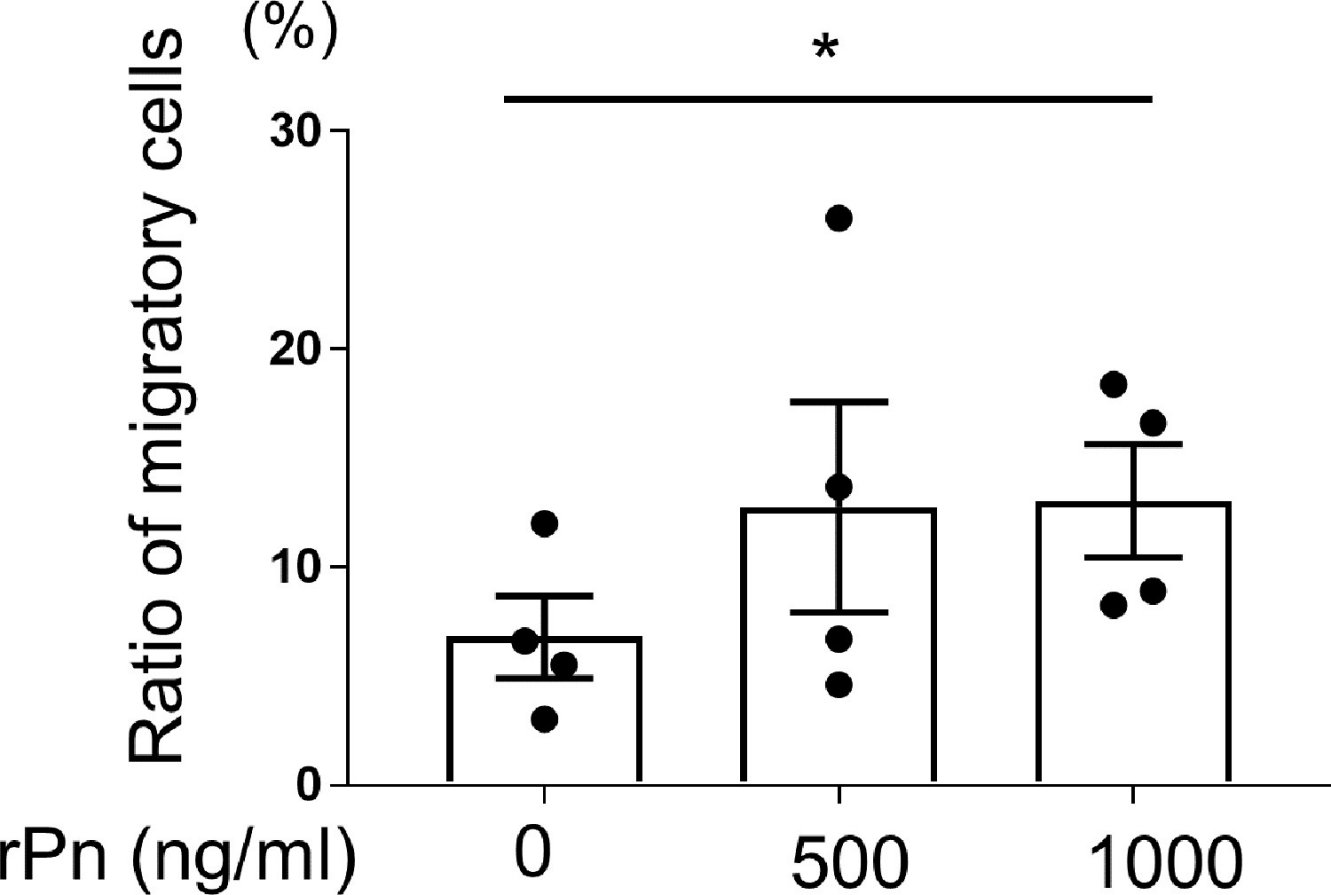
Periostin-mediated chemotaxis of monocytes. Monocytes from healthy controls were seeded in the upper compartment, and medium containing 500 or 1000 ng/mL of recombinant periostin (rPn) was added to the lower compartment of a Transwell. Two hours post-incubation, cells were collected from both compartments and manually counted. The graph displays the ratio of migrating monocytes. The results represent four independent experiments. Data are shown as a mean ± standard error of the mean (SEM). Statistical analysis was performed using one-way analysis of variance followed by Dunn’s test. n = 4, *P < 0.05.

Since both, the level of periostin and number of monocytes, were increased in the skin of SSc patients compared to that of HCs [2, 12, 23], the possibility of periostin-induced infiltration of monocytes in the skin was further evaluated. Periostin increased the percentage of migrating monocytes in a concentration-dependent manner. The ratio of monocytes that migrated toward the medium containing rPn (1000 ng/mL) was significantly higher than that of the medium without rPn (Fig 5).

## Discussion

The present study demonstrated the role of periostin in enhancing the expression of pro-fibrotic and pro-inflammatory factors associated with SSc pathology in monocytes and MDMs. To our knowledge, no study has reported the pro-fibrotic phenotypic role of periostin on monocytes and MDMs in the pathogenesis of SSc to date. In addition, we report the novel ability of periostin in promoting monocyte infiltration. Periostin has been reported to be associated with skin sclerosis in SSc patients [14]. Periostin induces fibroblast-mediated enhanced production of SSc-related ECM proteins [24]. Both periostin and monocytes are increased in the skin and peripheral blood of SSc patients [2, 12, 23], suggesting the involvement of periostin in recruiting circulating monocytes in the skin tissue. Thus, periostin stimulates monocytes to produce pro-fibrotic factors, thereby promoting fibrosis. Our results support previous findings suggesting periostin as an important factor in recruiting macrophages in the tumor microenvironment [25, 26]. It has also been reported that periostin plays an important role in inducing pericyte migration in the context of tissue fibrosis [27]. Taken together, these findings indicate that periostin may be involved in the promotion of various cell migrations.

In this study, we used RNA-seq and qPCR to show that PDGFRβ was upregulated in periostin-stimulated monocytes compared to its expression in control monocytes. The miRNA miR-30b is significantly downregulated in the serum of SSc patients compared to that of HCs. miR-30b is a negative regulator of PDGFRβ expression and inversely correlates with mRSS [28]. Furthermore, inhibition of PDGFRβ by transfection with miR-30b in skin fibroblasts from SSc patients significantly inhibits expression of αSMA and Col1A2. These findings suggest the involvement of PDGFRβ in the pathogenesis of fibrosis in SSc. Crenolanib-mediated inhibition of PDGFR signaling attenuates the expression of periostin and alleviates fibrosis in SSc patient fibroblasts and SSc preclinical models [29]. In addition, PDGF-BB induces expression of periostin through the PDGFRβ signaling pathway [30]. These reports, together with our results, imply a possible interaction between periostin and PDGFRβ. Activation of PDGFRβ promotes monocyte recruitment [31], suggesting its probable involvement in the promotion of periostin-mediated monocyte migration.

AGAP2-AS1, a long non-coding RNA (lncRNA), was elevated in periostin-stimulated monocytes. Genes that do not encode proteins account for approximately 98% of the entire genome, of which more than 200 bases are lncRNAs [32]. Recent studies have shown that approximately 80.4% of non-coding RNAs are responsible for DNA epigenetic modifications [33]. In SSc skin tissue, 676 non-coding RNAs were found to be DEGs compared to HCs, of which AGAP2-AS1 was among the first three antisense lncRNAs [34]. AGAP2-AS1 is involved in cell migration and promotes tumorigenesis by suppressing the tumor suppressors LATS2 and KLF2 [35]. However, its role in SSc remains to be elucidated.

In general, macrophages have been classified as classical (M1) or alternatively activated (M2) macrophages; M1 macrophages express specific cell surface markers, including CD80, while M2 macrophages mainly express macrophage scavenger receptors (CD163) and mannose receptor 1 (CD206) [36, 37]. In the present study, CD80^-^CD163^+^ MDM2 cells were significantly increased in SSc patients compared to HCs. This result was consistent with a previous study that reported elevated levels of CD163^+^ cells in CD14^+^ PBMCs from patients with SSc [2]. In addition, CD80^+^CD163^-^ MDM1 negatively correlated with mRSS, and CD80^-^CD206^+^ MDM2 positively correlated with mRSS. Accordingly, coculture of human PBMCs and B cells showed a positive correlation between B cell-induced CD206 expression and mRSS [6].

Several reports indicate a role of periostin in the polarization of macrophages in diseases other than SSc. The combination of TSLP and periostin increases Arg-1 levels, a known M2 marker, in scabies [38]. Contrary to our expectations, the results of the present study indicated that periostin reduced M2 macrophages in HCs. This difference may be attributable to the use of mouse peritoneal macrophages and because periostin was not the sole stimulant in previous studies. Inhibition of Arg-1 in mice overexpressing periostin [39] makes it challenging to discuss the function of periostin based solely on macrophage cell surface markers. In addition, our study suggested that periostin-induced changes in the polarization of MDMs might differ between HCs and SSc patients. The exact reason of this difference is not clear at present, but patients with SSc may be less responsive to rPn owing to pre-exposure to large amounts of periostin within the system. The differential effect of periostin on MDM polarity between HCs and SSc patients needs to be further investigated.

Most importantly, periostin upregulated pro-fibrotic factors in both MDM1 and MDM2. The pro-inflammatory cytokines IL-6 and TNF-α, which are produced at high levels by M1 macrophages [40], play an important role in SSc. Serum and skin IL-6 levels were significantly elevated in early SSc patients, and serum IL-6 positively correlated with mRSS [41, 42]. An interaction network between IL-6 and TGF-β involving fibroblasts and macrophages leads to fibrosis in SSc [43]. Serum TNF-α concentrations are elevated and correlate with pulmonary fibrosis and decreased vital capacity in SSc patients [44]. MCP-1 is highly expressed in the skin fibroblasts of SSc patients [45]. In a mouse model of bleomycin-induced dermal fibrosis, administration of anti-CCL2 neutralizing antibody reduced the degree of dermal fibrosis [46]. Clinically, CCL2 levels in the circulation have been reported to be a potential biomarker in SSc-related ILD [47]. Interestingly, in addition to M1 cytokines, M2-like pro-fibrotic factors, including TGF-β, were upregulated in rPn-stimulated MDM1 cells. This may be related to the concept of “beyond the M1/M2 paradigm” in SSc, which has been proposed in recent years [48]. EGR1, a transcription factor with increased expression in the skin and lungs of dcSSc patients, plays an important role in the TGF-β-mediated fibrotic response [49]. In a genome-wide study, EGR1 upregulated ECM-related genes, such as collagen and fibronectin, in human fibroblasts [50]. Periostin mediates fibroblast-induced enhanced TGF-β response and potentiates EGR1 and ECM proteins such as FN and αSMA [24]. Together with our findings that periostin enhanced SSc-related factors containing EGR1 in MDMs, periostin-targeted therapy for SSc seems to be an attractive option.

The small number of samples analyzed is a limitation of this study and three of the enrolled patients were treated with small doses of corticosteroids (<10 mg/day) and mycophenolate mofetil. In these patients, there were no changes in drug doses and disease status over a long period of time, including the study period. However, it is possible that the treatment status could affect the outcome. This study was based on *in-vitro* experiments performed in the presence of M-CSF. Thus, further studies are needed to clarify the effects of periostin on MDMs *in vivo*.

In conclusion, our results indicate the possible involvement of periostin in the pathogenesis of SSc by influencing monocytes and MDMs in promoting inflammation and fibrosis. Periostin and its downstream factors may be promising as future therapeutic targets for SSc.

## Supporting information

S1 Fig Representative experimental data on the purity of monocytes isolated from PBMCs. Flow cytometry was used to evaluate the expression of CD14-positive cells.
(DOCX)

S1 Table TaqMan® probe assay identification numbers. (DOCX)

S2 Table SYBR® RT-qPCR primer sequences. (DOCX)

S3 Table Differentially expressed gene (DEG) analysis results. There were 67 DEGs regulated by periostin.
(DOCX)
