# Supplementary material for: Periostin—An inducer of pro-fibrotic phenotype in monocytes and monocyte-derived macrophages in systemic sclerosis
Source: PLoS One. 2023 Aug 2;18(8):e0281881. doi: 10.1371/journal.pone.0281881 (PMC10395906; doi:10.1371/journal.pone.0281881)
Supplement: S1 Fig — Flow cytometry was used to evaluate the expression of CD14-positive cells. (DOCX) [file pone.0281881.s001.docx]

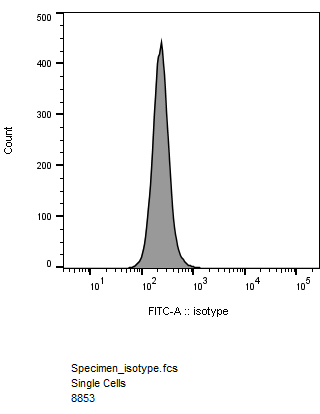

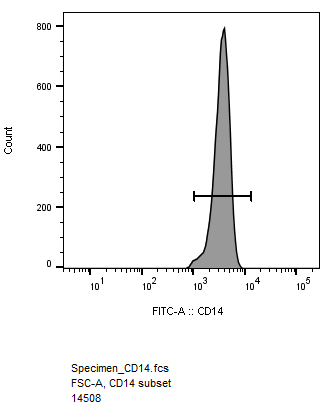


Supplementary Figure. S1

CD14-FITC

Cell counts

isotype

CD14

99.1%

Supplementary Figure. S1

A representative experimental data on the purity of monocytes isolated from PBMCs. Flow cytometry was used to evaluate the expression of CD14-positive cells.
