# Supplementary material for: Periostin—An inducer of pro-fibrotic phenotype in monocytes and monocyte-derived macrophages in systemic sclerosis
Source: PLoS One. 2023 Aug 2;18(8):e0281881. doi: 10.1371/journal.pone.0281881 (PMC10395906; doi:10.1371/journal.pone.0281881)
Supplement: S1 Table — (DOCX) [file pone.0281881.s002.docx]

Supplementary Table S1 TaqMan^®^ probe assay identification numbers

| TaqMan^®^ probes | Assay identification number |
| --- | --- |
| Human *ACTB* | Hs01060665_g1 |
| Human *ACTA2* | Hs00998193_m1 |
| Human *IL6* | Hs00985639_m1 |
| Human *EGR1* | Hs00152928_m1 |
| Human *TNFα* | Hs00174128_m1 |
| Human *CCL2* | Hs00234140_m1 |
| Human *TGFβ* | Hs00998133_m1 |
| Human *FN1* | Hs01549976_m1 |
| Human *PDGFRβ* | [Hs01019589_m1](https://www.thermofisher.com/taqman-gene-expression/product/Hs01019589_m1?CID=&ICID=&subtype=) |
