# Supplementary material for: Periostin—An inducer of pro-fibrotic phenotype in monocytes and monocyte-derived macrophages in systemic sclerosis
Source: PLoS One. 2023 Aug 2;18(8):e0281881. doi: 10.1371/journal.pone.0281881 (PMC10395906; doi:10.1371/journal.pone.0281881)
Supplement: S2 Table — (DOCX) [file pone.0281881.s003.docx]

Supplementary Table S2: SYBR^®^ RT-qPCR primer sequences

| Gene | SYBR^®^ qPCR primer sequence (5→3) | |
| --- | --- | --- |
|  | Forward | Reverse |
| Human *ACTB* | CTACAATGAGCTGCGTGTGGC | CAGGTCCAGACGCAGGATGGC |
| Human *AGAP2-AS1* | TACCTTGACCTTGCTGCTCTC | TGTCCCTTAATGACCCCATCC |
