# Supplementary material for: Periostin—An inducer of pro-fibrotic phenotype in monocytes and monocyte-derived macrophages in systemic sclerosis
Source: PLoS One. 2023 Aug 2;18(8):e0281881. doi: 10.1371/journal.pone.0281881 (PMC10395906; doi:10.1371/journal.pone.0281881)
Supplement: S3 Table — There were 67 DEGs regulated by periostin. (DOCX) [file pone.0281881.s004.docx]

| **Gene_**  **Symbol** | **periostin/ control.fc** | **test/control. raw.pval** |  | **Gene_**  **Symbol** | **periostin/ control.fc** | **test/control. raw.pval** |
| --- | --- | --- | --- | --- | --- | --- |
| **HOXA9** | -3.587671 | 0.01750902 |  | **LUZP6** | 2.540444 | 0.039968 |
| **KRASP1** | -3.566636 | 0.03235111 |  | **LOC102723458** | 2.555023 | 0.032738 |
| **RPL13AP24** | -3.502062 | 0.02572766 |  | **RFX4** | 2.56747 | 0.045496 |
| **RPS3P4** | -3.455455 | 0.0467153 |  | **CYP24A1** | 2.643003 | 0.048939 |
| **MRPL42P5** | -3.301158 | 0.0483166 |  | **PCLAF** | 2.670163 | 0.04762 |
| **LINC02861** | -3.229176 | 0.04087537 |  | **SETDB2-PHF11** | 2.691151 | 2.64E-06 |
| **SNURF** | -2.5982 | 0.00568849 |  | **CCL28** | 2.724097 | 0.046082 |
| **LOC101929018** | -2.437726 | 0.01232032 |  | **MSI1** | 2.735455 | 0.019567 |
| **CECR7** | -2.356877 | 0.02857536 |  | **RNF212** | 2.81809 | 0.044135 |
| **LINC01694** | -2.344383 | 0.03185479 |  | **SP2-AS1** | 2.827056 | 0.017955 |
| **CD1B** | -2.264695 | 0.01983178 |  | **PLEKHH2** | 2.850049 | 0.037495 |
| **HOMER3-AS1** | -2.238554 | 0.0146072 |  | **EME1** | 2.85186 | 0.03276 |
| **ANKHD1-EIF4EBP3** | -2.120776 | 0.00561062 |  | **PPIP5K1P1** | 2.857646 | 0.044808 |
| **KIF14** | 2.004981 | 0.02336748 |  | **DPY19L2P2** | 2.86601 | 0.010269 |
| **ARHGEF26** | 2.055884 | 0.0493439 |  | **LOC100506083** | 3.013357 | 0.028365 |
| **PNMA3** | 2.094702 | 0.02498311 |  | **CFL1P5** | 3.036148 | 0.030899 |
| **PDCD2L** | 2.118572 | 0.03528479 |  | **SHISAL1** | 3.095155 | 0.034949 |
| **LOC105374298** | 2.120825 | 0.04108518 |  | **RPL12P16** | 3.199055 | 0.007351 |
| **PDGFRB** | 2.120842 | 0.02232417 |  | **BRWD1-AS2** | 3.330071 | 0.038641 |
| **ARHGAP20** | 2.137064 | 0.03637466 |  | **CADM2** | 3.334065 | 0.04638 |
| **AGAP2-AS1** | 2.209608 | 0.04605281 |  | **MAP1LC3C** | 3.452969 | 0.023919 |
| **PPT2** | 2.230222 | 0.030471 |  | **TRH** | 3.536416 | 0.037739 |
| **RBM20** | 2.270903 | 0.01760227 |  | **TMEM249** | 3.608903 | 0.043165 |
| **FBXO17** | 2.279977 | 0.04896473 |  | **PDCL3P4** | 3.71433 | 0.043578 |
| **MKRN3** | 2.302081 | 0.04298849 |  | **NUDT10** | 3.818231 | 0.031976 |
| **LINC00565** | 2.327595 | 0.03069671 |  | **GRK5-IT1** | 3.867175 | 0.044241 |
| **HSF2BP** | 2.35362 | 0.02644913 |  | **SOX2-OT** | 3.916761 | 0.004517 |
| **GRHL3** | 2.356046 | 0.02294569 |  | **FOXO6** | 4.100827 | 0.040263 |
| **FAT3** | 2.361652 | 0.04365818 |  | **LOC105376689** | 4.677351 | 0.010614 |
| **P2RX5** | 2.414896 | 0.02653544 |  | **PIH1D2** | 5.030521 | 0.004506 |
| **FAM13C** | 2.419409 | 0.03426546 |  | **TGFB2-OT1** | 7.635935 | 0.030979 |
| **SIAH3** | 2.450475 | 0.04121111 |  | **PLA2G4B** | 8.932324 | 0.008087 |
| **FAM71F1** | 2.459182 | 0.03008397 |  | **ZNF625-ZNF20** | 24.74323 | 0.000523 |
| **VWCE** | 2.506931 | 0.04592851 |  |  |  |  |

Supplementary Table S3: Differentially expressed genes (DEGs) analysis results.

There are 67 DEGs regulated by periostin.
